# Editorial: Urban health: the next frontier for health policy and systems research

**DOI:** 10.3389/fpubh.2023.1212399

**Published:** 2023-06-15

**Authors:** Bruno Marchal, Joris Michielsen, Tolib Mirzoev, Ligia Paina, Sara Van Belle

**Affiliations:** ^1^Department of Public Health, Institute of Tropical Medicine, Antwerp, Belgium; ^2^London School of Hygiene and Tropical Medicine, London, United Kingdom; ^3^Department of International Health, Johns Hopkins Bloomberg School of Public Health, Baltimore, MD, United States

**Keywords:** urbanization, health, complex adaptive systems, governance, research priorities and challenges

In this Research Topic, we explore health policy and systems research at the nexus between urbanization and health. The global trend of urbanization will not abate in the near future and the related challenges are many. Low- and middle-income countries (LMICs) are observing a continued expansion of informal settlements, residential segregation, a double burden of life-long conditions and (re-emerging) infectious diseases. Important socio-ecological challenges relate to urban sprawl, unregulated economic activities, pollution, and environmental degradation. A central concern in LMICs is the impact of urbanization on equity and social inclusion (1, 2).

Over the last years, different research agendas for urban health have been published. Harpham called for a multi-level and multi-sectoral approach to research on the determinants of health in cities (3). Oni et al. presented a framework that included the “*community, experiential, environmental, and structural policy level*” (4). Vearey et al. argued that in order to achieve the SDGs, research and training in Africa should focus on intersectoral urban health literacy, urban governance for health, and participatory urban health planning processes (5). In 2022, the WHO presented four global research priorities for urban health, which all focused on generating better evidence (6). While interesting research has been carried out in the disciplines of urban studies (7), urban governance (8), sustainability (9), and climate change (10), studies on urbanization and health remain rare in the field of health policy and systems research (11). At the two most recent Global Symposia for Health Systems Research, the topic hardly featured on the programme (12). This led us to launch this Research Topic focusing on research, methodological developments, and innovative concepts related to the urbanization-health nexus.

Our view on urbanization and health is based on the conceptualization of cities as multi-dimensional ecosystems. Cities are shaped by the relationships among individuals and communities. Cities are best understood as complex in nature, engaging with multiple, connected, cross-sectoral “wicked problems”—examining health without taking these into account would be insufficient. The built environment, the institutional arrangements, the economic activities and the socio-ecological systems in which individual and collective dynamics occur impact directly on health and wellbeing, shape the capacity to respond to needs, and may contribute to constraining access to health services and resources by specific groups. Understanding the complex pathways that shape the health and wellbeing of urban residents is of key importance in building responsive urban health systems and ensuring equitable service delivery. Multi-sectoral action, adaptive governance and participatory approaches to health are of key importance.

In this Research Topic, Salgado et al. report on the use of dashboards to monitor environmental health in urban settings. The authors explored how end-users in Lisbon could be involved in the design of user-centered dashboards to better inform decision-makers. They adopted a complex systems co-design perspective to address “*the need to make available a wide range of built and natural environment indicators' data to address their multifactorial effects on health*.” The focus was on indicators for the built and the natural environment. The authors reinforce the argument that effective information systems that integrate evidence from across the multiple urban dimensions are central to sound urban health decision-making and planning. The next step could be to broaden the indicators in order to better account for the needs and expectations of the residents of Lisbon.

The occurrence of natural disasters and other large-scale emergencies represents another aspect of urban health that transcends sectors and dimensions. Li et al. present a model to help in deciding how to allocate resources during an emergency response. They developed a mobile emergency supplies allocation model to help decision-makers in distributing supplies as fast and efficiently as possible and applied it to the response to an episode of heavy rainfall in Zhengzhou (China). Their study uses modeling in management of urban emergencies and illustrates how such techniques can improve decision-making in crisis situations when uncertainty is high.

Having accurate and timely data in a polycentric urban decision-making space remains a big challenge (13). The papers of Almasi et al. and Banke-Thomas et al. report on the integration of geographical data in urban health policy and system analysis. Almasi et al. investigated the location of medical laboratories in Kermanshah (Iran) and its impact on access to laboratory services. The authors used a combination of GIS-based mapping of facilities and network analysis to map locations of laboratories and their accessibility for urban residents. They found coverage gaps in specific areas of the city, pointing to inequitable distribution of facilities and access problems. Banke-Thomas et al. investigated how, in big cities, the travel time of pregnant women requiring urgent care can be estimated using Google Maps data. The authors developed a method to estimate the travel time of women requiring Emergency Obstetric Care in Lagos (Nigeria). Next, they plan to co-create dashboards with urban health policymakers and data companies, which will combine data on travel time with data on the functionality of hospitals. Similar to Almasi et al., they highlight inequities related to the place of residence. Such approaches could contribute to a better understanding of how the urban advantage can become a disadvantage for those living in underserved areas (14).

Verdonck et al. present a community case study of “emergence,” describing how local health actors in neighborhoods with high ethnic diversity in Antwerp (Belgium) developed local initiatives for evidence building and information distribution during the COVID-19 wave of July 2020. Family practitioners considered that the contact tracing system set up by the Flemish government would be ineffective in their superdiverse neighborhoods. In < 2 weeks and in close collaboration with city and provincial authorities, they set up a successful alternative system of COVID coaches in support of contact tracing and self-isolation. This study illustrates how in situations of high uncertainty, national responses can and must be adapted to local contexts. Yet, this requires a strong learning culture, embedded in adaptive governance arrangements, and the importance of incorporating local innovations into broader policies and programs (15).

Unpacking the relationships between the social, political, economic, infrastructural, and ecological determinants of health in cities is key to understanding urban health. This requires multidisciplinary approaches and multi-actor knowledge production. Two papers address this methodological challenge. Dens et al. reflect on the use of drawings, which is often used in the discipline of urbanism to identify and analyse spatial patterns. The authors argue that drawings can be used in multidisciplinary research on the role of place in the transmission of infectious diseases in cities to “*(a) identify systemic relations within the spatial context, (b) facilitate integration of quantitative and qualitative data, and (c) guide the formulation of policy recommendations, informing public and urban health planning*.” They illustrate this in a study on malaria in Jimma (Ethiopia) and on tuberculosis and COVID-19 in Lima (Peru). Fang et al. addressed another intersecting set of challenges: aging, rapid urbanization, and increasing digitisation of social spaces and resources. This requires cities and communities to develop age-friendly community ecosystems that put intergenerational relationships at the center. The authors adopted a community-based participatory research approach and co-creation methods to co-create recommendations for research, policy, and practice. The paper illustrates how Bronfenbrenner's ecological systems theory provides a multi-level framework to assess how wellbeing is shaped an interplay of micro- and macro-level factors.

The papers included in this Research Topic present unique methodological, empirical, and practical contributions that speak to the complex nature of the urbanization-health nexus. Yet, they also show that research into urban inequities, residential segregation and social exclusion merits more attention. More research is also needed on the dynamic interplay between cities, the national and the global level regarding health. National development plans and urban policies shape cities. Cities have open boundaries through which people migrate continuously. Cities are also players in networked global markets and are hubs in global disease transmission patterns. Moreover, the interface between global warming and health calls for more research on intersectoral action for climate-resilient urban health systems.

This Research Topic shows that interesting and innovative research is being done at the still little explored frontier of urbanization and health. In these turbulent times, the challenges confronting urban (health) planners, managers, and researchers, as well as people, providers and communities are only amplified. The Health Policy and Health System Research community will need to rise up to contribute to making cities healthier for its people through more innovative studies.

## Author contributions

All authors contributed to the conceptualization, drafting, and final revision of this editorial.
